# Galectin-9 Induced by Dietary Probiotic Mixture Regulates Immune Balance to Reduce Atopic Dermatitis Symptoms in Mice

**DOI:** 10.3389/fimmu.2019.03063

**Published:** 2020-01-22

**Authors:** Han Wool Kim, Do Bin Ju, Yoon-Chul Kye, Young-Jun Ju, Cheol Gyun Kim, In Kyu Lee, Sung-Moo Park, In Soon Choi, Kwang Keun Cho, Seung Ho Lee, Sung Chan Kim, In Duk Jung, Seung Hyun Han, Cheol-Heui Yun

**Affiliations:** ^1^Department of Agricultural Biotechnology, Research Institute for Agriculture and Life Sciences, Seoul National University, Seoul, South Korea; ^2^Department of Biological Science, College of Medical and Life Sciences, Silla University, Busan, South Korea; ^3^Department of Animal Resources Technology, Gyeongnam National University of Science and Technology, Jinju, South Korea; ^4^Department of Nano-Bioengineering, Incheon National University, Incheon, South Korea; ^5^Department of Biochemistry, Institute of Cell Differentiation and Aging, College of Medicine, Hallym University, Chuncheon, South Korea; ^6^Laboratory of Dendritic Cell Differentiation and Regulation, Department of Immunology, School of Medicine, Konkuk University, Chungju, South Korea; ^7^Department of Oral Microbiology and Immunology, Dental Research Institute and Brain Korea 21 Plus Program, School of Dentistry, Seoul National University, Seoul, South Korea; ^8^Institute of Green Bio Science Technology, Seoul National University, Seoul, South Korea; ^9^Center for Food and Bioconvergence, Seoul National University, Seoul, South Korea

**Keywords:** probiotics, atopic dermatitis, dendritic cell, T cell balance, galectin-9

## Abstract

Probiotics can be an effective treatment for atopic dermatitis (AD), while their mechanism of action is still unclear. Here, we induced AD in mice with 2,4-dinitrochlorobenzene and administrated YK4, a probiotic mixture consisting of *Lactobacillus acidophilus* CBT LA1, *L. plantarum* CBT LP3, *Bifidobacterium breve* CBT BR3, and *B. lactis* CBT BL3. Then, we have validated the underlying mechanism for the alleviation of AD by YK4 from the intestinal and systematic immunological perspectives. Administration of YK4 in AD mice alleviated the symptoms of AD by suppressing the expression of skin thymic stromal lymphopoietin and serum immunoglobulin E eliciting excessive T-helper (Th) 2 cell-mediated responses. YK4 inhibited Th2 cell population through induce the proportion of Th1 cells in spleen and Treg cells in Peyer's patches and mesenteric lymph node (mLN). CD103^+^ dendritic cells (DCs) in mLN and the spleen were significantly increased in AD mice administered with YK4 when compared to AD mice. Furthermore, galectin-9 was significantly increased in the gut of AD mice administered with YK4. *In vitro* experiments were performed using bone marrow-derived DCs (BMDC) and CD4^+^ T cells to confirm the immune mechanisms of YK4 and galectin-9. The expression of CD44, a receptor of galectin-9, together with programmed death-ligand 1 was significantly upregulated in BMDCs following treatment with YK4. IL-10 and IL-12 were upregulated when BMDCs were treated with YK4. Cytokines together with co-receptors from DCs play a major role in the differentiation and activation of CD4^+^ T cells. Proliferation of Tregs and Th1 cell activation were enhanced when CD4^+^T cells were co-cultured with YK4-treated BMDCs. Galectin-9 appeared to contribute at least partially to the proliferation of Tregs. The results further suggested that DCs treated with YK4 induced the differentiation of naïve T cells toward Th1 and Tregs. At the same time, YK4 alleviated AD symptoms by inhibiting Th2 response. Thus, the present study suggested a potential role of YK4 as an effective immunomodulatory agent in AD patients.

## Introduction

Atopic dermatitis (AD) is one of the most common chronic inflammatory skin diseases seen mostly in children, although it can occur at any age. The main symptoms of AD are destruction of the stratum corneum and increased eczema and itching. AD is known to be caused by complex interactions between genetic factors and extrinsic allergens (1, 2). When AD occurs, scratching of the skin due to itching causes severe damage to the epidermal barrier of the skin followed by dysregulation of immunoregulatory proteins, such as interleukin (IL)-1, IL-25, IL-33, and thymic stromal lymphopoietin (TSLP), in skin epithelial cells (3, 4). These immunomodulatory proteins are known to initiate and promote T-helper (Th) 2 cell-mediated immune responses (5). Activated Th2 cells release IL-4, IL-13, and IL-31, and stimulate B cells to undergo isotype switching of immunoglobulin (Ig) M to IgE (6). The increased IgE production causes eosinophil accumulation in the dermis (7).

The immune imbalance induces an increase of inflammation in the skin and exacerbates the symptoms of AD. Moreover, AD patients have increased risk of other atopic diseases, including asthma, allergic rhinitis, and food allergies (8). Several drugs are currently under development to improve itch-associated symptoms of AD, and to show anti-inflammatory or epithelial barrier repair action. Monoclonal antibodies and protein inhibitors are also under investigation to block the cytokine and/or its receptor signaling pathway and thereby alleviate symptoms of AD (9). Although steroids are widely accepted as the first choice of anti-inflammatory drugs and intermittent use can reduce disease recurrence, long-term drug therapy with steroids should be avoided due to side effects, such as nausea, vomiting, diarrhea, skin thinning, and purpura (10). Therefore, alternative therapies, including herbs, plants, vitamins, and probiotics, have been studied (11, 12).

Probiotics are live microorganisms that provide beneficial effects on host health and disease prevention and treatment (13). Among various probiotics, *Lactobacillus, Bifidobacterium, Escherichia*, and *Saccharomyces* have been widely studied and commonly used in humans and animals (14). Ingested probiotics competed with harmful microorganisms to prevent pathogens from adhering to the epithelium in the intestine (15). Probiotics also enhanced the survival of intestinal epithelial cells and improved the barrier function, and production of immunomodulatory substances (16). Some probiotics reach to the lamina propria through M cells and interact with immune cells to regulate gastrointestinal immune system (17). Dendritic cells (DCs) in the lamina propria layer was known to be the main cell type that recognizes probiotics (18). DCs are one of the antigen-presenting cells that play a key role in bridging innate and adaptive immune responses (19). Specifically, DCs that were specialized for inhibiting inflammation, called tolerogenic DCs (tDCs), and CD103^+^ DCs played a similar role in the gastrointestinal area (20). CD103^+^ DCs inhibited naive CD4^+^ T cell differentiation into Th2 cells and, at the same time, induced the differentiation of regulatory T cells (Tregs) through the production of IL-10 and TGF-β (20). Recently, the effects of DCs primed by probiotics to control T cell responses have been reported (21, 22). *B. breve* Yakult induced the production of IL-10 in DCs through TLR2/MyD88 signal transduction and promoted the differentiation of Tregs (23). In addition, *L. plantarum* WCFS1 induced CD103^+^ DCs infiltration and generation of Tregs in the spleen (24). Duolac ATP, a mixture of four probiotic strains; i.e., *Lactobacillus casei* CBT LC5, *Lactobacillus plantarum* CBT LP3, *Lactobacillus rhamnosus* CBT LR5, and *Bifidobacterium lactis* CBT BL3, was reported to modulate the expression of costimulatory molecules of DCs and downregulate Th2 responses in an AD mouse model (25). Mixed probiotic strains of *Lactobacillus* and *Bifidobacterium* reduced the atopic dermatitis index in young AD patients (26) and an AD mouse model (27, 28). However, the mechanism of action of probiotics is only partially understood.

Galectins are soluble forms of lectin that exhibit specific binding activity for beta-galactoside sugars (29). Galectin-1, -2, -3, -4, and -9 are generally expressed in the gastrointestinal tract (30) and appear to be involved in the regulation of intestinal homeostasis and immunity (31). Especially, galectin-9 binds directly to CD44 and promotes Foxp3 expression of Tregs, which have been reported to suppress excessive Th2 responses (32). Indeed, recombinant galectin-9 treatment resulted in improved clinical and immunological symptoms of allergic diseases in mouse models (33). Recently, *Bifidobacterium breve* M-16V, used as a dietary supplement, was shown to induce elevation of serum galectin-9 in mice and humans (34). However, the precise mechanism by which galectin-9 modulates immune cells in AD therapy is not yet clear.

In the present study, we designed YK4, a mixture of four probiotic strains; i.e., *Lactobacillus acidophilus* CBT LA1, *L*. *plantarum* CBT LP3, *B*. *breve* CBT BR3, *and B*. *lactis* CBT BL3, which were selected based on their anti-inflammatory activities in bone marrow-derived DCs (BMDCs). This study was performed to evaluate the effects of YK4 on regulation of the intestinal and systemic immune systems using an AD mouse model in relation to galectin-9 expression and CD4^+^ T cells.

## Materials and Methods

### Animal

Female, 6- to 9-week old, BALB/c mice were purchased from Orient Bio (Gapyeong, South Korea). The mice were randomized and housed at 2–3 mice per individually ventilated cages under the controlled environment with a 12 h light-dark cycle. Water and diet are the principle of unlimited supply, but stopped 3 h before the administration of probiotics or phosphate buffered saline (PBS; Thermo Fisher Scientific, Waltham, MA, USA). All the experimental procedures were carried out in accordance with the Animal Use and Care Protocol approved by the Institutional Animal Care and Use Committee at Seoul National University, Seoul, Korea (Approval No. SNU-170428-1-1).

### Probiotics

Probiotic strains were obtained from Cell Biotech Co. Ltd (Gimpo, Korea) as a lyophilized powder form, containing 1 × 10^11^ CFU/g. YK4, used in the present study, is composed of four different strains of probiotics: *L. acidophius* CBT LA1 (KCTC11906BP), *L. plantarum* CBT LP3 (KCTC10782BP), *B. breve* CBT BR3 (KCTC12201BP), and *B. lactis* CBT BL3 (KCTC11904BP). The constituent probiotics of YK4 were mixed at the same weight ratio.

### Mouse With Atopic Dermatitis Model

**Figure 1:**
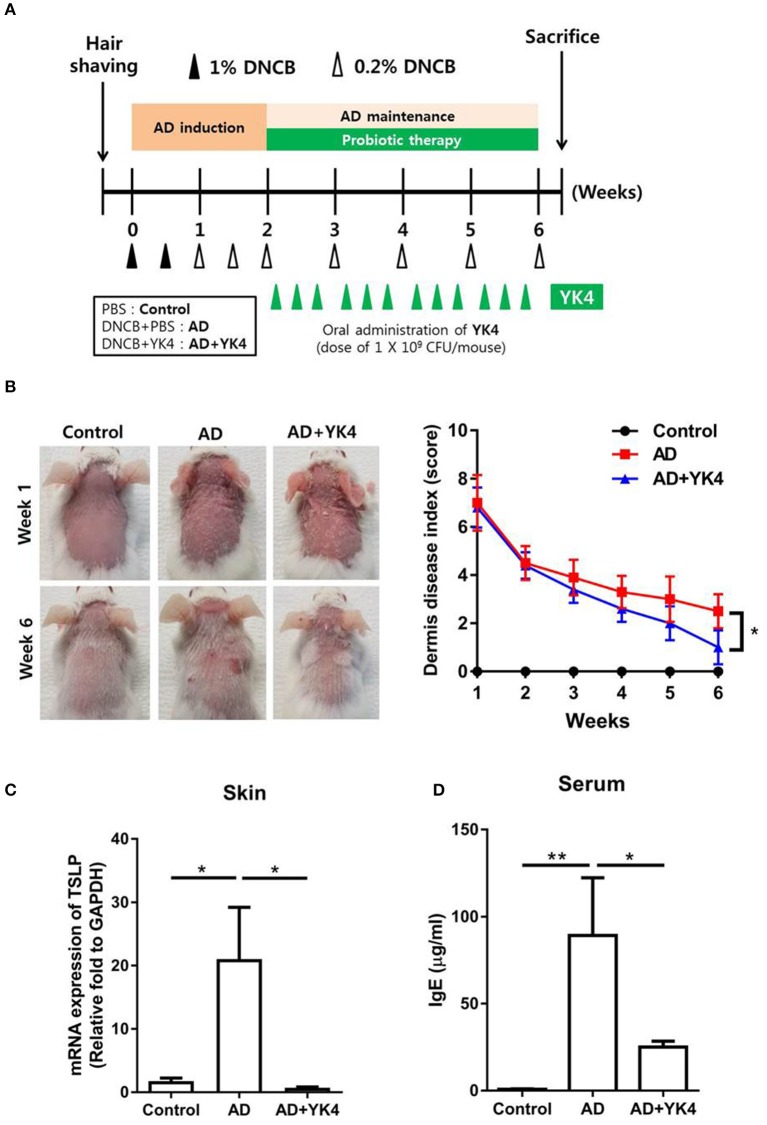
Amelioration of atopic dermatitis (AD)-like symptoms in mice treated with YK4. **(A)** Schematic diagram of 2,4-dinitrochlorobenzene (DNCB)-induced AD mouse model of AD-like skin lesions and oral administration of YK4 in BALB/c mice. DNCB was applied to the dorsal skin of BALB/c mice as described in the Materials and Methods. The mice were divided into three groups: (1) phosphate buffered saline (PBS) control (Control), (2) DNCB + PBS (AD), and (3) DNCB + YK4 (1 × 10^9^ CFU/day; AD + YK4). **(B)** AD-like skin lesions were evaluated by visual observation. Scoring of the dermatitis index was performed as described in the Materials and Methods. **(C)** Dorsal skin was also collected on week 6, and RNA was extracted for cDNA synthesis. qPCR was performed to examine thymic stromal lymphopoietin (TSLP) mRNA expression. Relative fold changes of target genes were compared with the housekeeping gene, GAPDH. **(D)** Blood samples were acquired and serum IgE levels were measured by ELISA. ^*^P < 0.05, ^**^P < 0.01 by one-way ANOVA with Tukey's multiple comparison test. Bars indicate means ± standard error of the mean (SEM).

The protocol to induce AD-like skin lesion in mouse model was modified from Lim et al. (35). Before the atopic induction, the dorsal hair of the mice were shaved and divided into three groups (*n* = 5 per group): (1) PBS control (Control), (2) 2,4-dinitrochlorobenzene (DNCB; Sigma-Aldrich, St. Louis, MO, USA) + PBS (AD), and (3) DNCB + YK4 (1 × 10^9^ CFU/day; AD + YK4). In order to induce the atopy, skin was topically sensitized with 1% DNCB that was dissolved in acetone-olive oil (3:1) twice for the first week, and 0.2% DNCB for three times in the second week. Once atopy was induced, 0.2% DNCB was applied for the maintenance purpose at once a week for 3–6 weeks. While maintaining the atopy, mice were administered a total of 12 times with PBS (200 μl/day) or YK4 (1 × 10^9^ CFU/200 μl/day) using feeding needle for three times a week. At the end of the treatment, the mice were anesthetized by using CO_2_ (Figure 1A). Then, blood samples were taken from the mice and dorsal skin, intestines, spleen, mesenteric lymph node (mLN) and Peyer's patches (PP) were extracted.

### Dermatitis Index

The severity of dermatitis score was evaluated once a week after DNCB treatment. Scores of 0 (none), 1 (mild), 2 (moderate), and 3 (severe) were examined for each of the four symptoms: erythema/hemorrhage, scarring/dryness, edema and excoriation/erosion. The sum of the individual scores indicating clinical severity was taken as the dermatitis score (36).

### Sample Preparation

Blood samples were collected by retro-orbital bleeding into heparinized tubes. The sera were then collected by centrifugation for 10 min at 1,500 × g and stored at −80°C, until further use. The mice were sacrificed at the end of the experiment. Spleen and large intestine were collected and the length was measured to determine the visual examination and inflammatory responses. Dorsal skin, and small and large intestines were collected for the mRNA expression of galectin-9. Spleen, mLN and PP were taken and rinsed with PBS. Then, they were placed on the 70 μm cell strainer (BD Biosciences) and ground using the back of the syringe to make single cells. Red blood cells were depleted using RBC-lysis buffer (Sigma-Aldrich) and the cells were stained with the proper combination of antibodies labeled with fluorescence and examined by using flow cytometry (FACScanto, BD Biosciences).

### Generation and Culture of BMDCs

BM cells were isolated from femurs of mice. Red blood cells were depleted using RBC-lysis buffer (Sigma-Aldrich) and BM cells were cultured in a complete RPMI with 20 ng/ml GM-CSF (Creagene, Seongnam, Korea). The complete RPMI was composed of RPMI-1640 supplemented with 10% fatal bovine serum, 20 mM HEPES, 1 mM sodium pyruvate, 220 nM 2-mercaptoethanol, 100 μg/ml gentamicin (all from Sigma-Aldrich). BM cells were seeded at 3 × 10^6^ cells/well in a 6-well plate in 3 ml media, and then 2 ml of fresh media was added at day 3 and 5. At day 6, a half of the culture supernatant was carefully discarded, and 3 ml of fresh media was added. At day 7, suspended BM cells were harvested and sorted by CD11c MicroBeads UltraPure kit (Miltenyi Biotec, Bergisch Gladbach, Germany). Suspended CD11c^+^ BM cells (i.e., BM-derived DCs, BMDCs) were seeded at 2 × 10^5^ cells/well in 96-well plate and stimulated with LA1 (2 × 10^6^ CFU/well), LP3 (2 × 10^6^ CFU/well), BR3 (2 × 10^6^ CFU/well), BL3 (2 × 10^6^ CFU/well), YK4 (2 × 10^6^ CFU/well), 100 ng/ml of Lipopolysaccharide (LPS, Sigma-Aldrich) and 1 μg/ml of recombinant mouse galectin-9 (R&D Systems, Minneapolis, MN, USA) in a complete RPMI. After the incubation for 24 h, the supernatant was collected for the examination of cytokine concentration.

### *In vitro* CD4^+^ T Cell Stimulation

CD4^+^ T cells were isolated from mLN from wild type mice using mouse CD4^+^ T lymphocyte enrichment Set (BD Biosciences, San Jose, CA, USA). The CD4^+^ T cells were labeled with CellTrace™ Violet (CTV) Cell Proliferation Kit (Thermo Fisher Scientific). CD4^+^ T cells (2 × 10^5^ cells/well) were co-cultured with BMDCs (2 × 10^4^ cells/well) that had been treated with YK4 (2 × 10^5^ CFU/well) and/or galectin-9 (1 μg/ml), on anti-CD3 mAbs (BD Biosciences)-coated (2 μg/ml) 96-well plate. After the incubation for 72 h, the cells were examined for proliferation of Foxp3^+^CD4^+^ T cells by using flow cytometry and the supernatant was collected and examined for IL-4, IL-10, IL-17, and IFN-γ concentration by using ELISA.

### RNA Isolation and qPCR

Total RNA was isolated from dorsal skin and intestines by TRIzol® reagent (Thermo Fisher Scientific). One microgram of RNA was reverse-transcribed in a 20 μl reaction containing random primers (500 μg/ml), dNTP (10 mM), 5× first strand buffer, Dithiothreitol (0.1 M), Superscript III enzyme (200 U/μl) and RNase inhibitor (10 U/μl) (all from Thermo Fisher Scientific). The quantitative PCR (qPCR) was performed with the iQ SYBR Green Supermix (Bio-Rad Laboratories, Hercules, CA, USA) on the LightCycler 480 Real-Time PCR System (Roche, Mannheim, Germany). This was then used to calculate the relative amounts of target mRNA in test samples. Quantities of all targets in test samples were normalized to the corresponding GAPDH levels. Primers mouse galectin-9 (forward: 5′-CAG CAC CCC TGG ACA GAT GT-3′, reverse: 5′-ATG GAC TTG GAC GGG TAA AGC-3′), mouse TSLP (forward: 5′-TAC TAT ACT CTC AAT CCT ATC CCT G-3′, reverse: 5′-ACT TCT TGT GCC ATT TCC TG-3′), mouse GAPDH (forward: 5′-CTC CAC TCA CGG CAA ATT CA-3′, reverse: 5′-GCC TCA CCC CAT TTG ATG TT-3′) were synthesized from Bioneer Inc. (Daejeon, Korea).

### Enzyme-Linked Immunosorbent Assay (ELISA)

The immunological response of the mice with DNCB-induced AD was monitored by measuring the levels of serum IL-4, IL-10, IL-12p40, IL-17, TGF-β, IFN-γ (all from R&D Systems) and IgE (BD Biosciences). Using the ELISA DuoSet kit (R&D Systems), mouse IL-6, IL-10 and IL-12p40 were measured in stimulated BMDCs supernatant, and IL-4, IL-10, IL-17 and IFN-γ were measured in stimulated T cells supernatant *in vitro*. Briefly, 96-well microplate (Thermo Fisher Scientific) was pre-coated with 100 μl/well of capture antibody. After blocking with 1% BSA for 1 h at room temperature, 100 μl/well of supernatant along with the standard solution diluted in diluent buffer was added and incubated for 2 h at room temperature. After the wash with PBS for three times, 100 μl/well of biotinylated detection antibody was added and incubated for 2 h at room temperature. Then, the plate was washed with PBS for three times and 100 μl of streptavidin-HRP in PBS was added. After the incubation for 20 min at room temperature, tetramethylbenzidine (TMB, Merck Millipore, Burlington, MA, USA) was added to develop the color and then the reaction was stopped by adding 50 μl of 2M H_2_SO_4_. The absorbance at wavelength 450 nm was measured by a microplate reader (Molecular Devices, San Jose, CA, USA).

### Phenotypic and Functional Examination of Immune Cells by Using Flow Cytometry

In order to examine the activation status of the cells, BMDCs were treated with YK4 and/or galectin-9 for 24 h at 37°C. The cells were stained with anti-mouse CD44-FITC, CD86-FITC, PD-L1-PE, MHC II-PE-cy7, OX40L-APC, CD11c-APC (all from BD Biosciences) for 20 min at 4°C in the dark. To test the change of Tregs, CTV-labeled CD4^+^ T cells cultured with YK4 and/or galectin-9-treated BMDCs for 3 days were stained with anti-mouse CD25-FITC and CD4-PE (BD Biosciences). After surface staining, CD4^+^ T cells were fixed and stained with anti-mouse Foxp3-APC mAb (Biolegend, San Diego, MA, USA) using Foxp3 Fix/Perm Buffer Set (Biolegend). *In vivo* examination, spleen, mLN, and PP were isolated from the mice and single cells were prepared. Population changes of DCs were examined after the staining with combination of anti-mouse CD103-bv421, MHC II-PE-cy7, and CD11c-APC (all from BD Biosciences) for 20 min at 4°C in the dark. To examine the preferential subpopulation of CD4^+^ T cells, splenocytes, and mononuclear cells from mLN and PP were stimulated with 50 ng/ml of phorbol 12-myristate-13-acetate (PMA) and 750 ng/ml of ionomycin (Sigma-Aldrich) in the presence of brefeldin A (Sigma-Aldrich) for 4 h. Then, the cells were stained with appropriate combination of anti-mouse CD4-bv605, IFN-γ-PE, IL-4-bv605, and IL-17-APC-cy7 mAb (all from Biolegend). The cells were washed and the expression of fluorescence was examined using a FACS Canto II (BD Biosciences). All flow cytometric data acquired were analyzed with FlowJo software (Tree Star, Ashland, OR, USA).

### Statistical Analysis

The levels of significance for the comparison between *in vivo* samples were determined by Tukey's multiple comparison test by using GradPad InStat software (Ver 5.01, GraphPad). The data were expressed as the mean ± SEM. *In vitro* data were evaluated using Student's *t*-test, and *p* < 0.05 was considered as statistically significant.

## Results

### Amelioration of AD in Mice Treated With YK4

To investigate the therapeutic properties of YK4 *in vivo*, we established a mouse model with AD-like skin lesions. As shown in Figure 1A, the mice were sensitized with DNCB for 2 weeks and then given phosphate buffered saline or 1 × 10^9^ CFU of YK4 intragastrically. The AD group showed severe atopic symptoms including erythema/hemorrhage, edema, excoriation/erosion, and scaling/dryness. Oral administration of YK4 significantly ameliorated the severity of AD-like skin lesions compared to the AD group (Figure 1B). It has been suggested that inflammatory responses caused by AD may affect other organs. For example, strong Th2 inflammation causes intestinal contraction and splenomegaly (37, 38). No significant changes were found on visual or physical examination of the AD mice based on the length of the spleen and large intestine at the end of the feeding period (Supplementary Figures 1A,B). It has been suggested that the expression of TSLP in keratinocytes is one of the main features of AD (39). To investigate the induction of skin inflammation, we measured TSLP mRNA expression in the skin. The results showed that TSLP in the skin of the AD group was significantly increased, whereas it remained at a normal level in the AD group treated with YK4 (Figure 1C). Excessive production of serum IgE is a typical characteristic of AD symptoms in accordance with the induction of a Th2-type immune response (2, 40). The results of this study showed that serum IgE levels were significantly increased in the AD group compared to the control group, whereas the AD group administered YK4 showed significantly lower IgE (Figure 1D). These results suggested that YK4 effectively inhibited the expression of TSLP and IgE, thereby ameliorating the symptoms of AD.

### YK4 Administration Induced a Decrease in the Th2 Response Coincident With an Increase in Tregs *in vivo*

**Figure 2:**
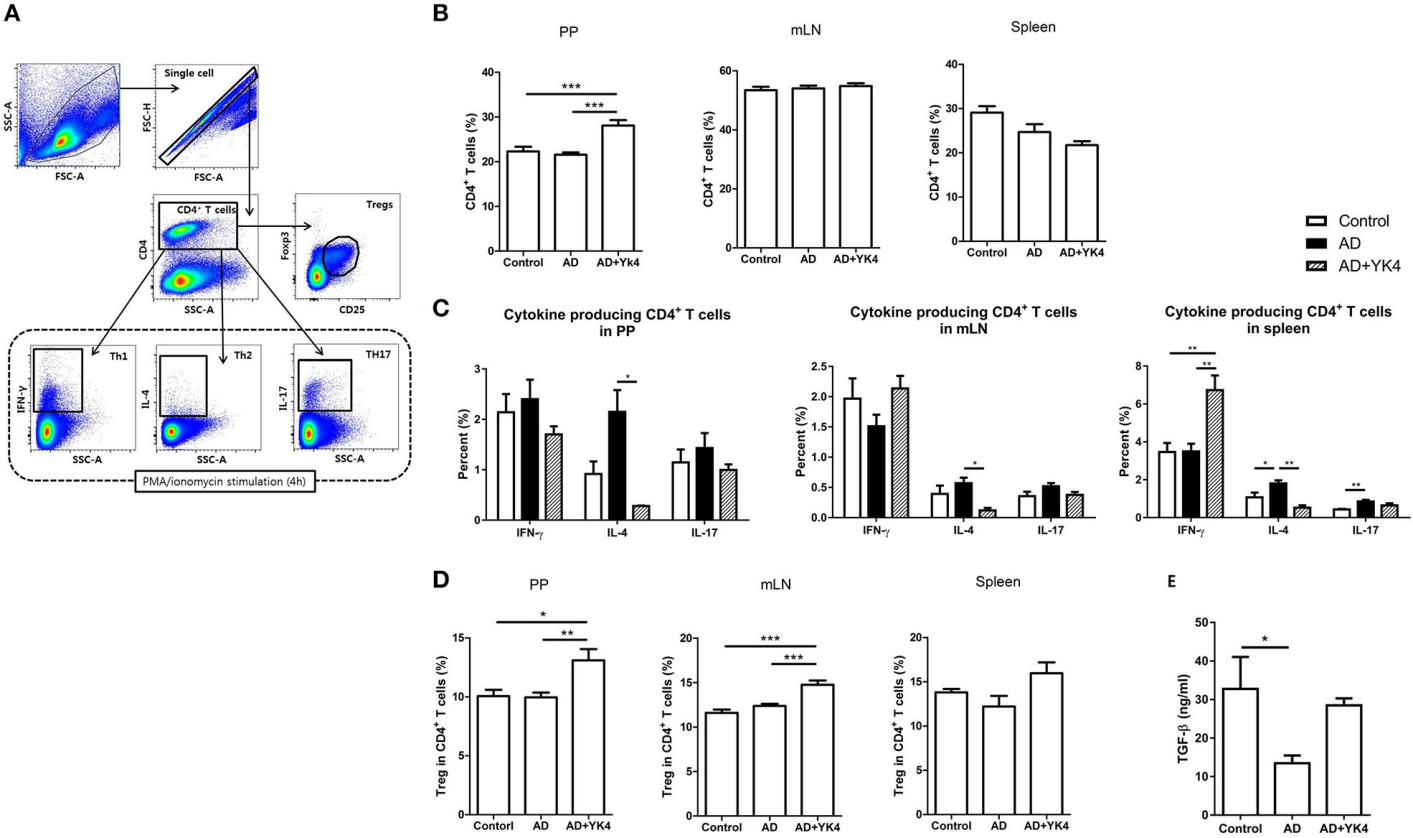
Characterization of CD4^+^ T cells from Peyer's patches (PP), mesenteric lymph nodes (mLN), and the spleen of DNCB-sensitized BALB/c mice treated with YK4. DNCB-induced AD mice were fed YK4, and PP, mLN, and the spleen were collected on week 6. Single cells from each tissue were used for characterization of CD4^+^ T cells. **(A)** Gating strategy for CD4^+^ T cell subtypes. **(B)** The percentages of total CD4^+^ T cells from PP, mLN, and the spleen were examined by flow cytometry. **(C)** Single cells from PP, mLN, and the spleen were stimulated with PMA/ionomycin in the presence of brefeldin A for 4 h. IFN-γ-, IL-4-, and IL-17-producing CD4^+^ T cells from PP, mLN, and the spleen were examined following intracellular staining by flow cytometry. **(D)** The percentages of Foxp3^+^CD25^+^CD4^+^ T cells from PP, mLN, and the spleen were examined by flow cytometry. **(E)** Blood samples were taken and serum TGF-β levels were measured by ELISA. Data are representative of at least three experiments. *P < 0.05, **P < 0.01, ***P < 0.001 by one-way ANOVA with Tukey's multiple comparison test. Bars indicate means ± SEM.

AD is triggered by hypersensitivity of the Th2 response, which can be overcome by rebalancing CD4^+^ T cell subsets (25). To determine whether YK4 administration affects intestinal and systemic T cell responses, subpopulations of CD4^+^ T cells in mLN, PP, and the spleen obtained using the gating strategy shown in Figure 2A were examined in AD mice with/without YK4 administration. The results showed that the proportion of CD4^+^ T cells in PP was significantly increased in AD mice administered YK4, but no changes were observed in mLN and the spleen (Figure 2B). Next, we examined the other subtypes of helper T cells by testing the intracellular expression of interferon (IFN)-γ, IL-4, and IL-17 in CD4^+^ T cells. The ratio of Th1 to Th17 cells did not change, while the ratio of Th2 cells decreased significantly in PP and mLN from AD mice administered YK4. On the other hand, the Th1 response in the spleen was increased in AD mice administered YK4 (Figure 2C), suggesting that it potentially counteracted the decrease in the Th2 response. Th2-induced allergic reactions are known to be suppressed by Tregs (41). Therefore, we examined the changes in populations of Tregs in PP, mLN, and the spleen of AD mice administered YK4. The Tregs populations in PP and mLN were increased in AD mice administered YK4 compared to control and AD groups, while no changes were found in the spleen (Figure 2D).

Cytokine production in atopic disease is one of the indicators used to indirectly examine the systemic immune response. For example, IL-10 and transforming growth factor (TGF)-β affect the differentiation and function of Tregs (42). Therefore, we measured serum cytokines to assess their possible roles in the relationship between YK4 and T cell responses. The results showed that the levels of serum IFN-γ, IL-4, IL-10, and IL-17 were below the limits of detection in all groups (data not shown). However, the serum TGF-β level was significantly decreased at the onset of AD, and recovered following administration of YK4 (Figure 2E). These results indicated that YK4 induces a decrease in the Th2-type response coincident with an increase in Tregs in the intestine and induction of serum TGF-β expression.

### YK4 Administration Induced an Increase in CD103^+^ Dendritic Cells *in vivo*

**Figure 3:**
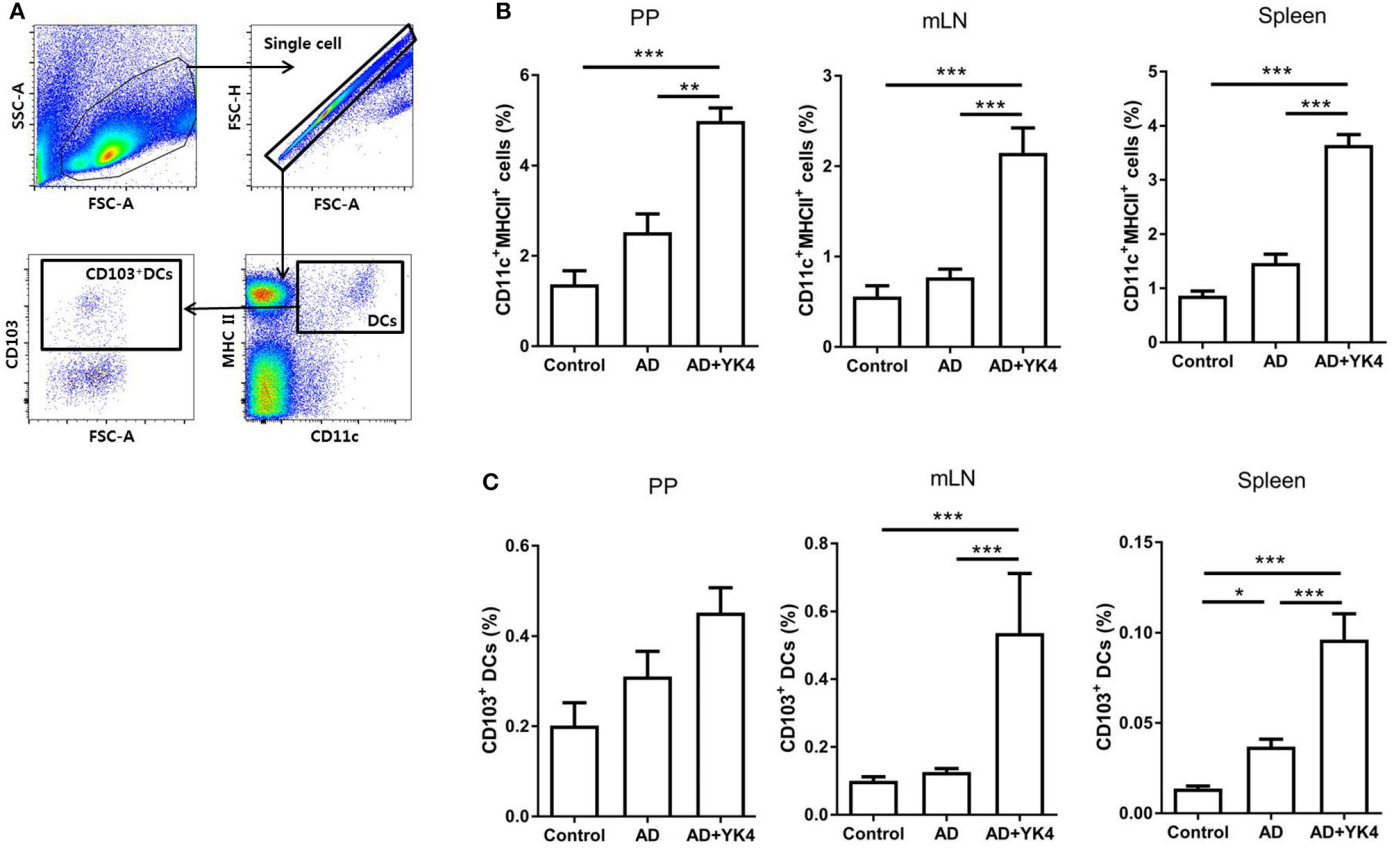
Composition of dendritic cells (DCs) from PP, mLN, and the spleen in DNCB-sensitized BALB/c mice treated with YK4. DNCB-induced AD mice were fed YK4, and PP, mLN, and the spleen were collected on week 6. Single cells from each tissue were used to examine the proportion of DCs. **(A)** Gating strategy for DC subtypes. The percentages of **(B)** CD11c^+^MHC II^+^ DCs and **(C)** CD103^+^ DCs from PP, mLN, and the spleen were examined by flow cytometry. Data are representative of at least three experiments. *P < 0.05, **P < 0.01, ***P < 0.001 by one-way ANOVA with Tukey's multiple comparison test. Bars indicate means ± SEM.

DCs are among the most important antigen presenting cells for the differentiation of naïve T cells into their specific subsets. In particular, CD103^+^ DCs are known to induce Tregs differentiation in the gastrointestinal tract (43). In the present study, the proportions of CD11c^+^MHCII^+^ and CD103^+^ DCs in AD mice administered YK4 were examined using the gating strategy shown in Figure 3A. The proportions of DCs in PP, mLN, and spleen were significantly increased in AD mice administered YK4 in comparison to AD mice (Figure 3B). Moreover, the increases in CD103^+^ DCs in mLN and the spleen were pronounced (Figure 3C). Taken together, these results suggested that CD103^+^ DCs were increased in AD mice administered YK4.

### Galectin-9 in the Intestine Appears to Be Associated With Alleviation of AD Symptoms

**Figure 4:**
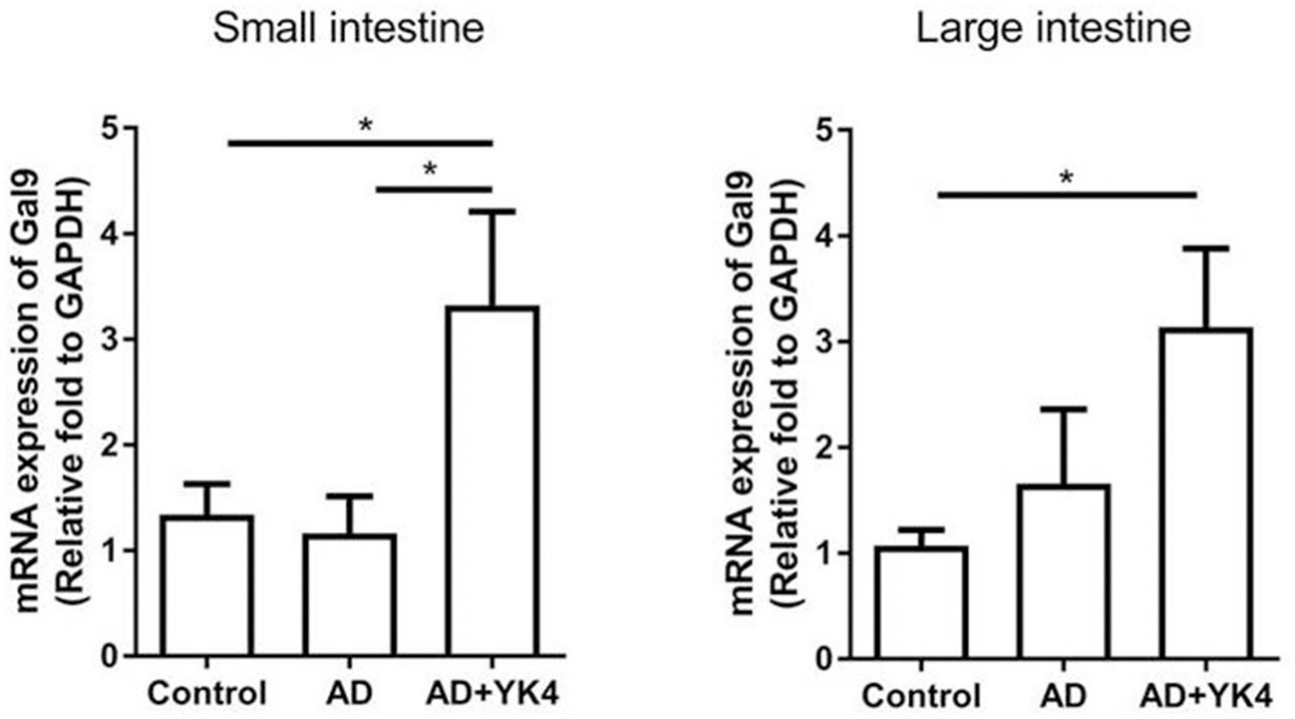
Expression of galectin-9 in the intestine of DNCB-sensitized mice treated with YK4. DNCB-induced AD mice were fed YK4. The small and large intestine were collected on week 6, and RNA was extracted for cDNA synthesis to examine the expression of galectin-9 mRNA (Gal9). Relative fold changes of target genes were compared with the housekeeping gene, GAPDH. *P < 0.05 by one-way ANOVA with Tukey's multiple comparison test. Bar indicates mean ± SEM.

Galectin-9 is known to regulate the immune response via modulation of DCs with sequential differentiation of Tregs (44, 45). We examined the expression of galectin-9 in the intestine to examine whether it plays a role in the suppression of AD-like symptoms. The results showed that there were no differences in the expression of galectin-9 in the small and large intestine between control and AD groups (Figure 4). However, galectin-9 expression was significantly increased in AD mice administered YK4 (Figure 4). These results suggested that YK4 induces an increase in galectin-9 in the intestine, suggesting the potential involvement of galectins in changes of CD103^+^ DCs and Tregs in AD mice.

### YK4 and Galectin-9 Effectively Induced Regulatory Immune Responses by BMDCs

**Figure 5:**
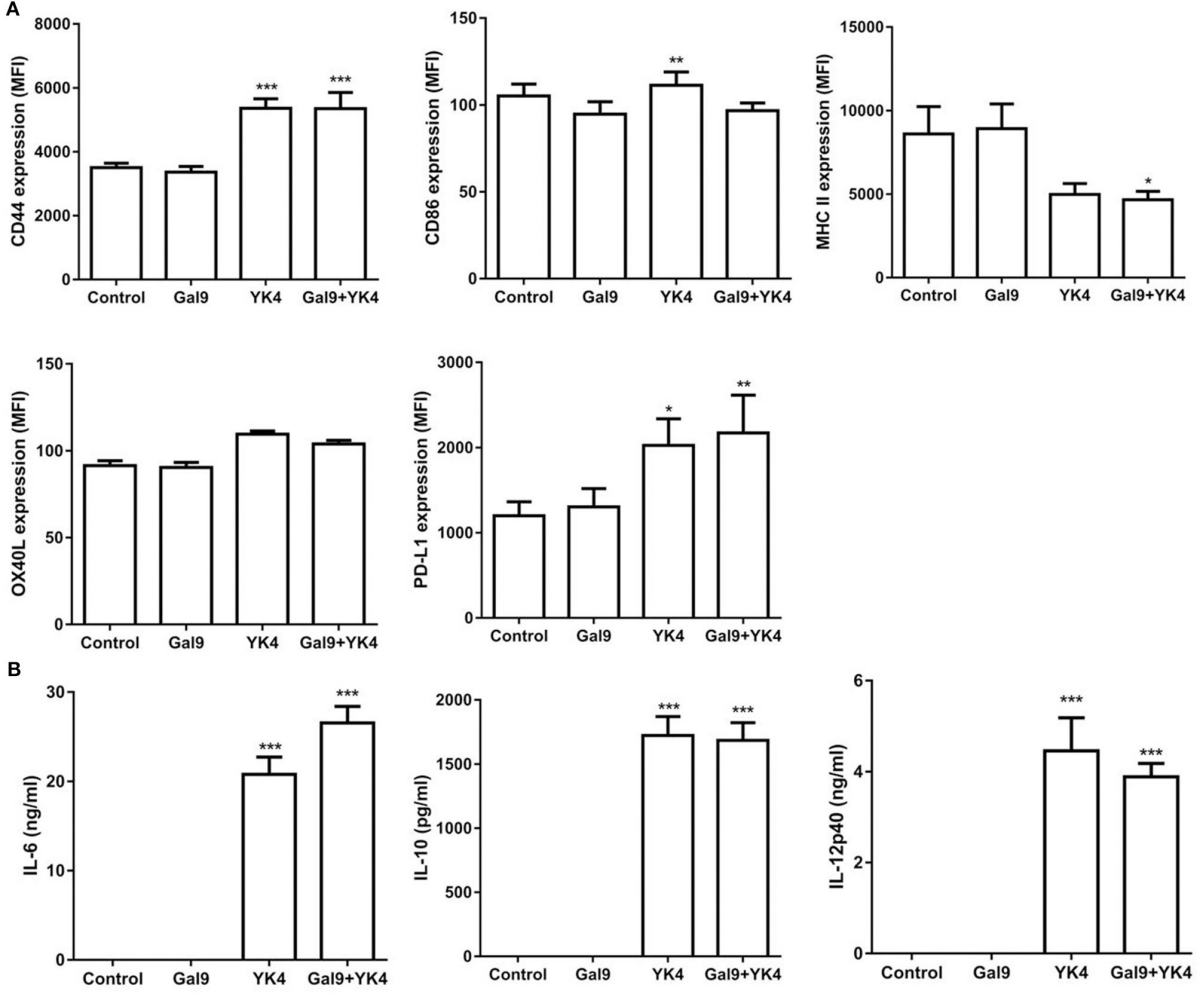
Changes of regulatory molecules in bone marrow-derived DCs (BMDCs) treated with YK4 and/or galectin-9. BMDCs were treated with galectin-9 and/or 2 × 10^6^ CFU of YK4 for 24 h. **(A)** The expression levels of CD44, CD86, MHC II, OX40L, and PD-L1 on BMDCs were measured by flow cytometry. **(B)** The expression levels of cytokines in the supernatants were measured by ELISA. Data are representative of at least three experiments. *P < 0.05, **P < 0.01, ***P < 0.001 compared to the non-treated control group (Control) by Student's *t*-test.

Next, we examined how YK4 and galectin-9 affect DCs. First, we investigated whether YK4 and/or galectin-9 affects the survival of BMDCs. The results showed that the survival of BMDCs was affected by neither YK4 at doses lower than 2 × 10^7^ CFU (Supplementary Figure 2A) nor by galectin-9 at doses lower than 10 μg/ml (Supplementary Figure 2B). Based on these results, YK4 and galectin-9 were used at 2 × 10^6^ CFU and 1 μg/ml, respectively. First, expression of the galectin-9 receptor, CD44, on BMDCs was examined. While treatment with galectin-9 alone did not alter the expression of CD44 on BMDCs, significant increases were observed when the cells were treated with YK4 alone or together with galectin-9 (Figure 5A). Galectin-9-treated BMDCs showed surface expression of CD86 similar to untreated cells, while its level slightly increased by YK4 treatment. MHC II expression was decreased when the cells were treated with both YK4 and galectin-9 (Figure 5A). Expression of OX40L, a ligand involved in induction of Th2 cell differentiation, was unaffected by treatment of BMDCs with galectin-9 and/or YK4 (Figure 5A). Expression of PD-L1, which is responsible for the immunosuppressive response, was increased following treatment of the cells with both YK4 and galectin-9 (Figure 5A). Immunomodulatory cytokines were examined in the culture supernatants of BMDCs treated with YK4 and/or galectin-9. The results showed that the expression level of the proinflammatory cytokine IL-6 was increased in BMDCs treated with YK4, and this was further increased by treatment with both YK4 and galectin-9 (Figure 5B). Expression of anti-inflammatory cytokine, IL-10 was also increased in BMDCs treated with YK4, while galectin-9 treatment with/without YK4 did not affect its expression (Figure 5B). IL-12p40, the expression of which was increased in BMDCs treated with YK4, was slightly decreased in cells treated with YK4 along with galectin-9 (Figure 5B). Taken together, these results suggest that YK4 makes BMDCs functionally tolerant. Moreover, galectin-9 further increased the tolerance of YK4-treated BMDCs.

### YK4 and Galectin-9 Induced Proliferation of Tregs and Increased Immunomodulatory Cytokine Levels

**Figure 6:**
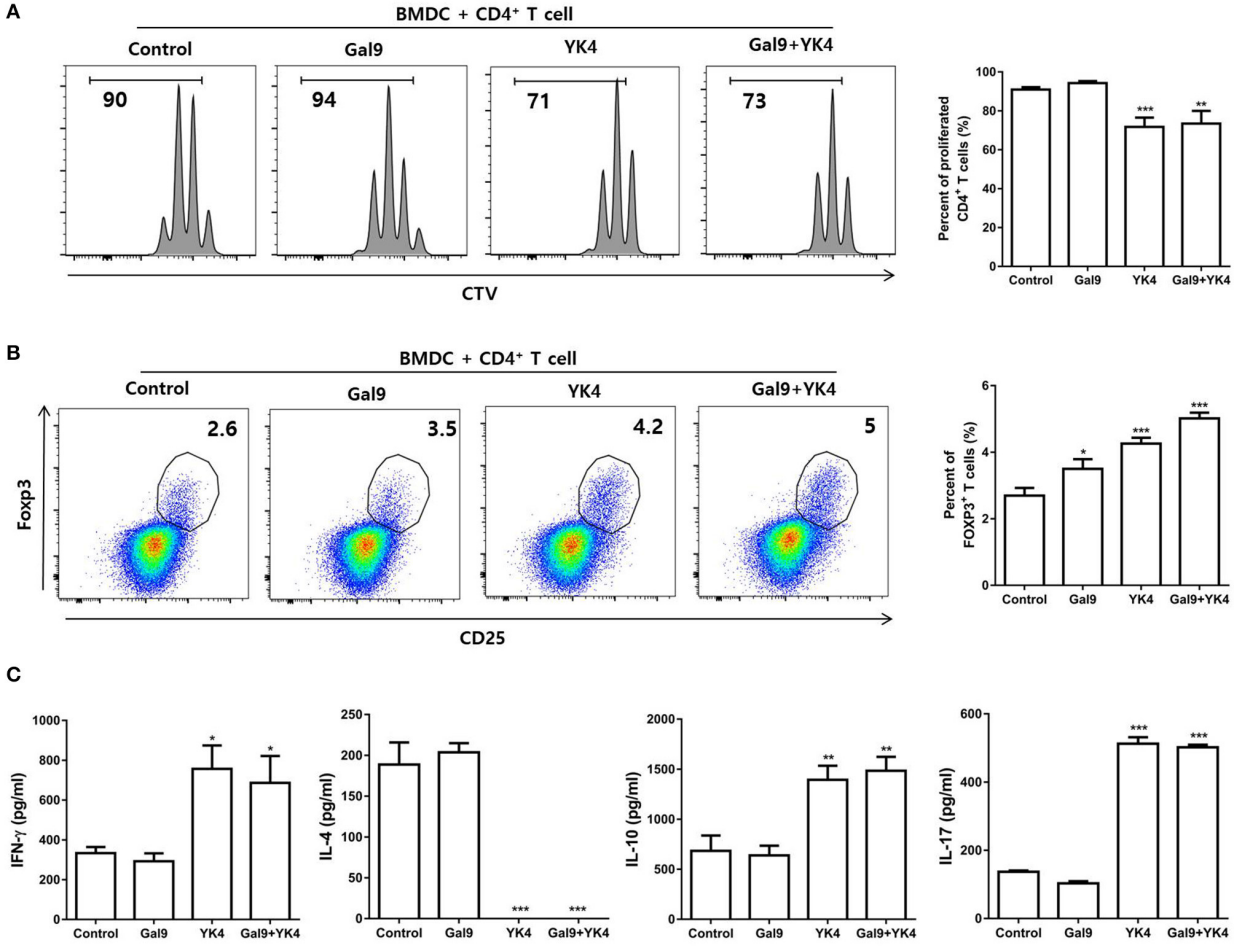
BMDCs treated with YK4 and galectin-9 promote Treg proliferation and immunomodulatory cytokine production. BMDCs treated with YK4 and/or galectin-9 were co-cultured with CD4^+^ T cells for 3 days on anti-CD3 mAb-coated plates. The percentage of **(A)** proliferated total CD4^+^ T cells and **(B)** proportion of CD4^+^CD25^+^Foxp3^+^ T cells among CD4^+^ T cells were analyzed by flow cytometry. At the same time, supernatants were harvested and examined for **(C)** the production of IFN-γ, IL-4, IL-10, and IL-17 in CD4^+^ T cells by ELISA. Data are representative of at least three experiments. *P < 0.05, **P < 0.01, ***P < 0.001 compared to the non-treated control group (Control) by Student's *t*-test.

Tregs are the main cells involved in induction of tolerance and suppression of excessive immune response. We investigated whether BMDCs treated with YK4 and/or galectin-9 could induce Treg proliferation. CD4^+^ T cells were isolated from mLN and co-cultured on anti-CD3 antibody-coated plates with BMDCs that had been treated with YK4 and/or galectin-9. The results showed that treatment of BMDCs with galectin-9 failed to inhibit proliferation of CD4^+^ T cells, while YK4-treated BMDCs showed inhibition of CD4^+^ T cell proliferation (Figure 6A). Next, we examined the proportion of Tregs among CD4^+^ T cells following co-culture with BMDCs. The proportion of Tregs increased when co-cultured with YK4-treated BMDCs, and a greater increase was observed in culture with YK4 and galectin-9-treated BMDCs (Figure 6B). To further confirm the activation of CD4^+^ T cells, the levels of immunomodulatory cytokines were investigated in the supernatants of CD4^+^ T cells co-cultured with YK4 and/or galectin-9-treated BMDCs. Galectin-9 alone did not affect the expression of immunomodulatory cytokines in CD4^+^ T cells (Figure 6C). However, the expression of IL-4 was reduced, while the levels of IL-10 and IL-17 were significantly increased in CD4^+^ T cells treated with YK4 and galectin-9 (Figure 6C). These results suggested that YK4 and galectin-9-treated BMDCs promoted IL-10 production and Tregs proliferation resulting in inhibition of other CD4^+^ T cells functions.

## Discussion

Probiotics are known to maintain health status upon ingestion in humans and animals by regulating intestinal immune homeostasis (46, 47). Despite numerous studies on allergies, the protective effects and precise mechanism of action of probiotics remain unclear. In the present study, we examined the regulatory effects against AD of YK4, a probiotic mixture with potent anti-inflammatory properties. Furthermore, we elucidated the mechanism underlying the anti-AD effect by showing that YK4 and galectin-9 regulate CD4^+^ T cells through DCs and Tregs.

In general, when the skin epidermal barrier is damaged by AD, TSLP is produced by keratinocytes. TSLP promotes activation of DCs and induces the expression of OX40L on the surface of DCs (48). The activated DCs together with IL-4 induce differentiation of naive T cells to form Th2 cells, which in turn contribute to the generation of IgE in B cells (49, 50). As expected, in the present study, DNCB-induced AD mice showed skin barrier disruption and inflammation due to excessive Th2 response followed by increases in serum IgE and skin TSLP levels. Previous studies have indicated that probiotics inhibit TSLP and IgE production to control AD symptoms (51). Intestinal microbiota plays a fundamental role in the induction and function of the host immune system (52). In particular, probiotics are known to affect the intestinal microbiota and to control disease propensity by the modulation of functional property of immune cells (53). It has been suggested that certain probiotics induce specific subsets of the intestinal CD4^+^ T cell population and alleviate AD symptoms. For example, the probiotic mixture, Duolac ATP, is known to downregulate Th2 cells in mLN and systemically inhibit Th2 responses (25). Treatment with *L*. *plantarum* WCFS1 was shown to downregulate Th2 cells in the intestinal lamina propria (54). Similarly, in the present study, the numbers of IL-4-producing Th2 cells decreased not only in mLN and PP but also in the spleen of DNCB-induced AD mice fed YK4. These findings suggested that YK4 might inhibit TSLP and IgE production by controlling intestinal and systemic Th2 cells.

Several strategies have been proposed for managing Th2 responses in AD, including the preferential differentiation of CD4^+^ T cells towards Th1 cells. Th1-type responses are known to counteract Th2 responses, thereby inhibiting the progression of inflammation in AD (55). Cytokines play an essential role in determining the direction and function of CD4^+^ T cells and their differentiation by activating STAT (56). IL-12, which is predominantly produced by antigen-presenting cells and phosphorylates STAT4 in CD4^+^ T cells, plays an important role in the differentiation of naïve T cells to Th1 cells (57). Phosphorylated STAT4 promotes T-bet expression and induces the production of IFN-γ, which again activates STAT1 in CD4^+^ T cells to further stabilize T-bet (57). This inhibits the expression of GATA3 and prevents differentiation of CD4^+^ T cells into IL-4-producing Th2 cells (58). *Lactobacillus paracasei* MoLac-1 has been shown to induce IL-12 production in DCs via Toll-like receptor (TLR) 9 signaling to inhibit the Th2 response (59, 60). In addition, the probiotic mixture, Duolac ATP, activated T-bet to induce a Th1 response and inhibited GATA3, thereby blocking the Th2 response in an AD mouse model (25). The levels of T-bet and GATA3 expression were reduced in the small intestine following treatment with *L*. *plantarum* WCFS1 in the mouse small intestinal lamina propria (54). The *Weissella cibaria* strain, WIKIM28, decreased IL-4 levels without affecting IFN-γ in peripheral lymph node cells (35). These results suggest that probiotics may contribute to a specific T cell response depending on the type and specific combination. In the present study, YK4 and its components induced IL-12 expression in DCs (Supplementary Figure 3A), which would directly affect the activity of STAT4 in CD4^+^ T cells. With this mechanism, YK4 increased IFN-γ and suppressed IL-4 production in CD4^+^ T cells through DCs. Furthermore, YK4 induced activation of Th1 cells, which produced IFN-γ and inhibited IL4-producing Th2 cells in the spleen of AD mice. These results suggest that YK4 induces differentiation of Th1 cells and downregulates the Th2 response in the spleen through IL-12 production by DCs.

Another mechanism underlying suppression of the Th2 response would involve Tregs-dependent regulation of unwanted inflammatory responses (61). Like other CD4^+^ T cells, Tregs are also stabilized and differentiated though specific cytokine signals, such as TGF-β and IL-10 (62). TGF-β activates Smad3 in Tregs and stabilizes Foxp3, which induces TGF-β and IL-10 production at the same time as differentiation, thereby suppressing unwanted immune responses (63). Certain probiotics can induce these immunosuppressive cytokines and promote the generation of Foxp3^+^ Tregs. For example, administration of WIKIM28 induced the differentiation of Tregs and the production of IL-10 in AD mice (35). Our study also showed that YK4 and its components probiotics induced IL-10 expression in BMDCs (Supplementary Figure 3B). Moreover, YK4 increased the expression of TGF-β, which was reduced in AD, to normal levels and increased the population of intestinal Foxp3^+^ Tregs. As a mechanism of action, tolerogenic DCs (tDCs) are essential to induce the differentiation of Tregs (64). Intake of probiotics can be recognized by TLRs on CD103^+^ DCs, which are abundant in the small intestine (65). Then, activated tDCs produce suppressive cytokines, including IL-10 and TGF-β, and cell-surface inhibitory molecules, PD-L1 and PD-L2 (66). Certain probiotics, such as *Bifidobacterium bifidum* PRI1, have been shown to increase the number of CD103^+^ DCs in the colonic lamina propria (67). Indeed, oral administration of YK4 in the present study induced upregulation of CD103^+^ DCs in mLN. Furthermore, DCs treated with YK4 induced increases in IL-10 and PD-L1 expression. Interestingly, when YK4-treated DCs were co-cultured with CD4^+^ T cells, the proportion of Tregs increased markedly. These results suggest that YK4 induces the activity of tDCs and affects the differentiation and activity of intestinal Tregs, thereby suppressing the intestinal Th2 response.

Probiotics may directly regulate immune cells, but they can also regulate the Th2 response indirectly by activating intestinal epithelial cells (68). When intestinal epithelial cells are activated by probiotics, effector molecules, including chemokines and galectins, are produced (69). In particular, galectin-9 is known to play a role in regulating AD through modulation of the immune response (70). The ingestion of dietary supplements with *B*. *breve* M-16V increased galectin-9 levels in intestinal epithelial cells thus preventing allergic symptoms (34). Similarly, we also observed an increase in the expression of intestinal galectin-9 in AD mice administered YK4. Galectin-9 binds directly to carbohydrate moieties of IgE to prevent IgE-antigen complex formation and mast cell degranulation. Moreover, galectin-9 binds to CD44 of DCs preventing their maturation and activation by inhibiting STAT1 activation (44). In the present study, galectin-9 did not directly affect the activity of DCs. However, when galectin-9 and YK4 were both administered, the expression of IL-12 was decreased in DCs. The activation of STAT1 by YK4 stimulation should precede the inhibitory effect of galectin-9 in DCs. The inhibition of IL-12 through galectin-9 also modulates the YK4-induced Th1 response. Galectin-9 is also known to bind CD44 on Tregs and phosphorylate Smad3 to stabilize Foxp3 followed by secretion of IL-10 and TGF-β (31). A number of reports have suggested a direct impact of galectin-9 on T cells; however, these studies did not consider the importance of the T cell–DC interaction for the best outcome of T cell activity. Thus, in the present study, we used BMDCs rather than anti-CD28 monoclonal antibodies to activate CD28 on CD4^+^ T cells. The results showed that galectin-9 did not cause BMDCs to become tolerogenic. Instead, treatment with galectin-9 together with YK4 induced proliferation of Tregs and promoted IL-10 production. TLR stimulation, such as by YK4 treatment, in DCs may be essential for galectin-9 to affect CD4^+^ T cells.

In this study, we investigated how YK4 affects immune cells in conjunction with cytokines and galectin-9 in the intestinal and systemic immune organs. YK4 effectively inhibited the Th2 response, but the intestinal and systemic immune responses were not the same. The intestines were immunologically tolerated compared with systemic immune organs (71). These fundamental environmental differences suggest that YK4 induced Th1 responses in the systemic organs and Tregs in the intestinal organs. In addition, metabolites of YK4 such as short-chain fatty acid may have a more direct effect on intestinal immune cells. Of course, further researches are needed to verify if the immune factors produced in the gut affect systemic organs. It has been reported that probiotics ingested would not settle effectively in the intestine, and its efficacy disappeared when ingestion is stopped (72). Therefore, further research is mandatory to determine if YK4 could influence the intestinal flora. It is important to mentioned that we are in need for comparative studies on females and males. For instance, under the certain condition, females have more CD4^+^ T cells than males (73). In addition, females are more sensitive to activation of TLRs and cytokines receptors under the influence of X chromosome (74). Male mice, on the other hand, have more numbers of Tregs than females and produce more IgE, especially during the fetal development via yet to be known mechanism (75).

**Figure 7:**
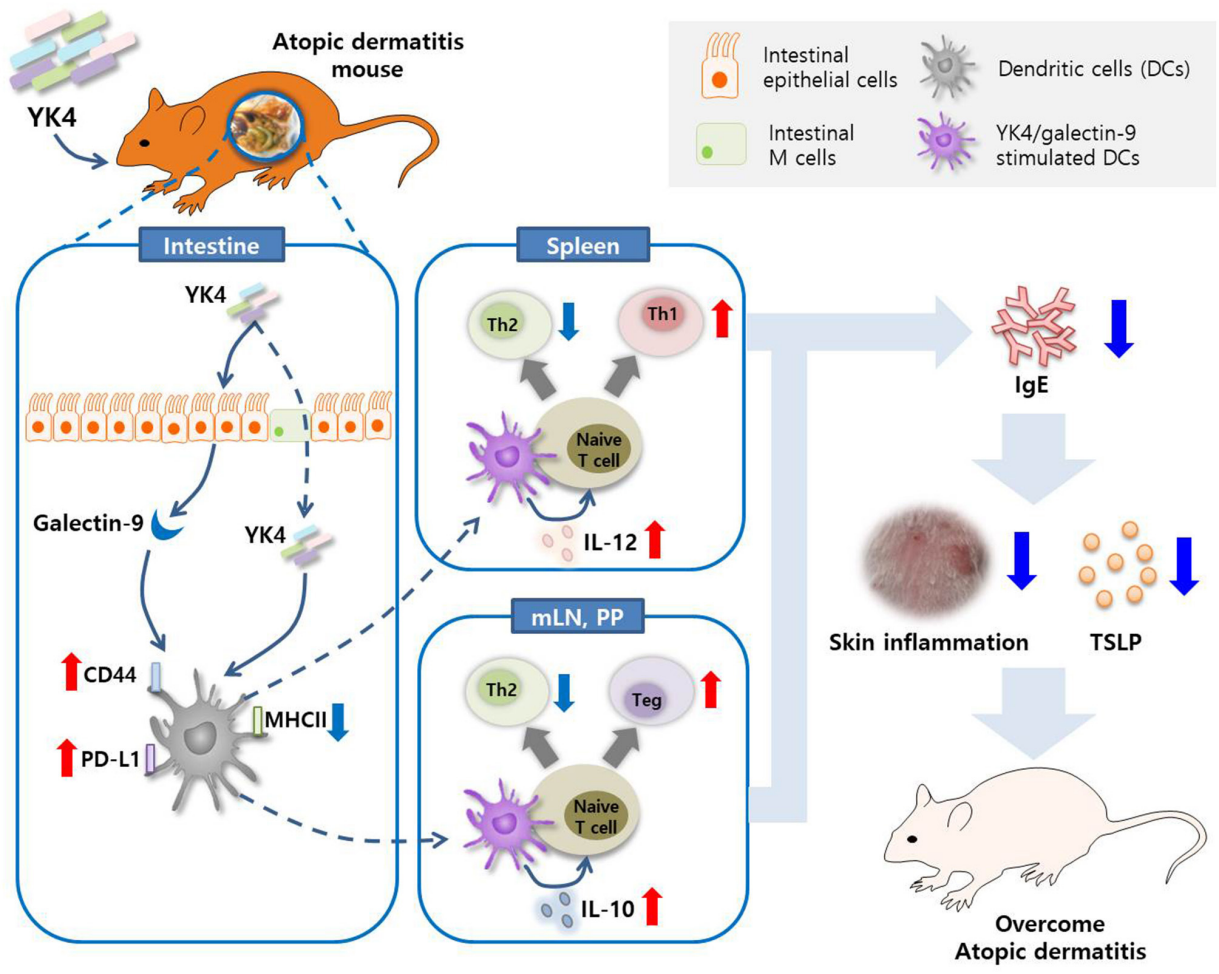
Graphical summary. The probiotic mixture, YK4, induced the production of galectin-9 in the intestine in a mouse model of AD. Galectin-9 together with YK4 induced the expression of PD-L1 and CD44 and the production of IL-10 and IL-12 in DCs. These DCs inhibited Th2 cell proliferation by induction of intestinal Tregs and splenic Th1 cell proliferation, thereby alleviating atopic symptoms.

In summary, our results showed that YK4 regulated intestinal galectin-9. Galectin-9, along with YK4, regulates the expression of IL-10 and IL-12 in DCs. These DCs, particularly CD103^+^ DCs, induced naïve T cell differentiation in Th1 and Tregs and inhibited Th2 responses (Figure 7). Taken together, these observations suggest that the probiotic mixture, YK4, has therapeutic potential to prevent AD symptoms and may act as an immunomodulator for AD patients.

## Data Availability Statement

All datasets generated for this study are included in the article/Supplementary Material.

## Ethics Statement

The animal study was reviewed and approved by the Institutional Animal Care and Use Committee at Seoul National University, Seoul, Korea (Approval No. SNU-170428-1-1).

## Author Contributions

C-HY conceived and designed the experiments. HK, DJ, Y-CK, Y-JJ, CK, IL, and S-MP performed the experiments and analyzed the data. C-HY and HK wrote the draft of the manuscript. SH, IJ, SK, SL, KC, and IC contributed to analyses and directed the experimental work together with a critical revision of the manuscript. All authors discussed and finalized the manuscript.

### Conflict of Interest

The authors declare that the research was conducted in the absence of any commercial or financial relationships that could be construed as a potential conflict of interest.

## Supplementary Material

Supplementary Figure 1 YK4 treatment does not induce inflammation in the spleen and large intestine. DNCB-induced AD mice were fed YK4. The **(A)** spleen and **(B)** large intestine were isolated on week 6. The length was measured by visual inspection to determine the inflammatory response.

Supplementary Figure 2 Apoptosis of BMDCs treated with YK4 and galectin-9. BMDCs (2 × 10^5^ cells) were treated with various concentration of **(A)** YK4 and **(B)** galectin-9 for 24 h. Then, the percentage of apoptotic cells (the propidium iodide-positive fraction) was measured. H_2_O_2_ treatment was used as a positive control. A histogram of the results is shown. Data are representative of at least three experiments. ^*^*P* < 0.05, ^**^*P* < 0.01, ^***^*P* < 0.001 compared to the non-treated control group (Control) using Student's *t*-test.

Supplementary Figure 3 Changes of cytokine secretion in BMDCs treated with each component of YK4. BMDCs were treated with 100 ng/ml of LPS or 2 × 10^6^ CFU of (YK4, BR3, BL3, LA1, LP3) for 24 h. The expression of **(A)** IL-12 and **(B)** IL-10 in the supernatants was measured by ELISA. Data are representative of at least three experiments. ^**^*P* < 0.01, ^***^*P* < 0.001 compared to the YK4-treated group (Control) using Student's *t*-test.
