# Supplementary figures and images for: Galectin-9 Induced by Dietary Probiotic Mixture Regulates Immune Balance to Reduce Atopic Dermatitis Symptoms in Mice
Source: Front Immunol. 2020 Jan 22;10:3063. doi: 10.3389/fimmu.2019.03063 (PMC6987441; doi:10.3389/fimmu.2019.03063)

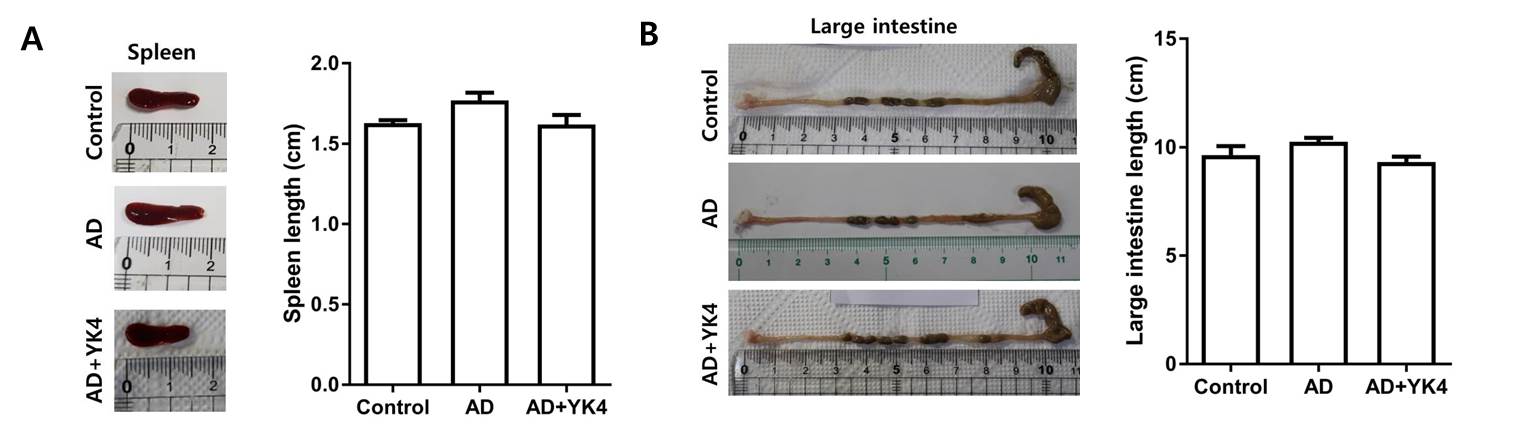

Supplement: Supplementary Figure 1 — YK4 treatment does not induce inflammation in the spleen and large intestine. DNCB-induced AD mice were fed YK4. The (A) spleen and (B) large intestine were isolated on week 6. The length was measured by visual inspection to determine the inflammatory response. [file Image_1.JPEG]

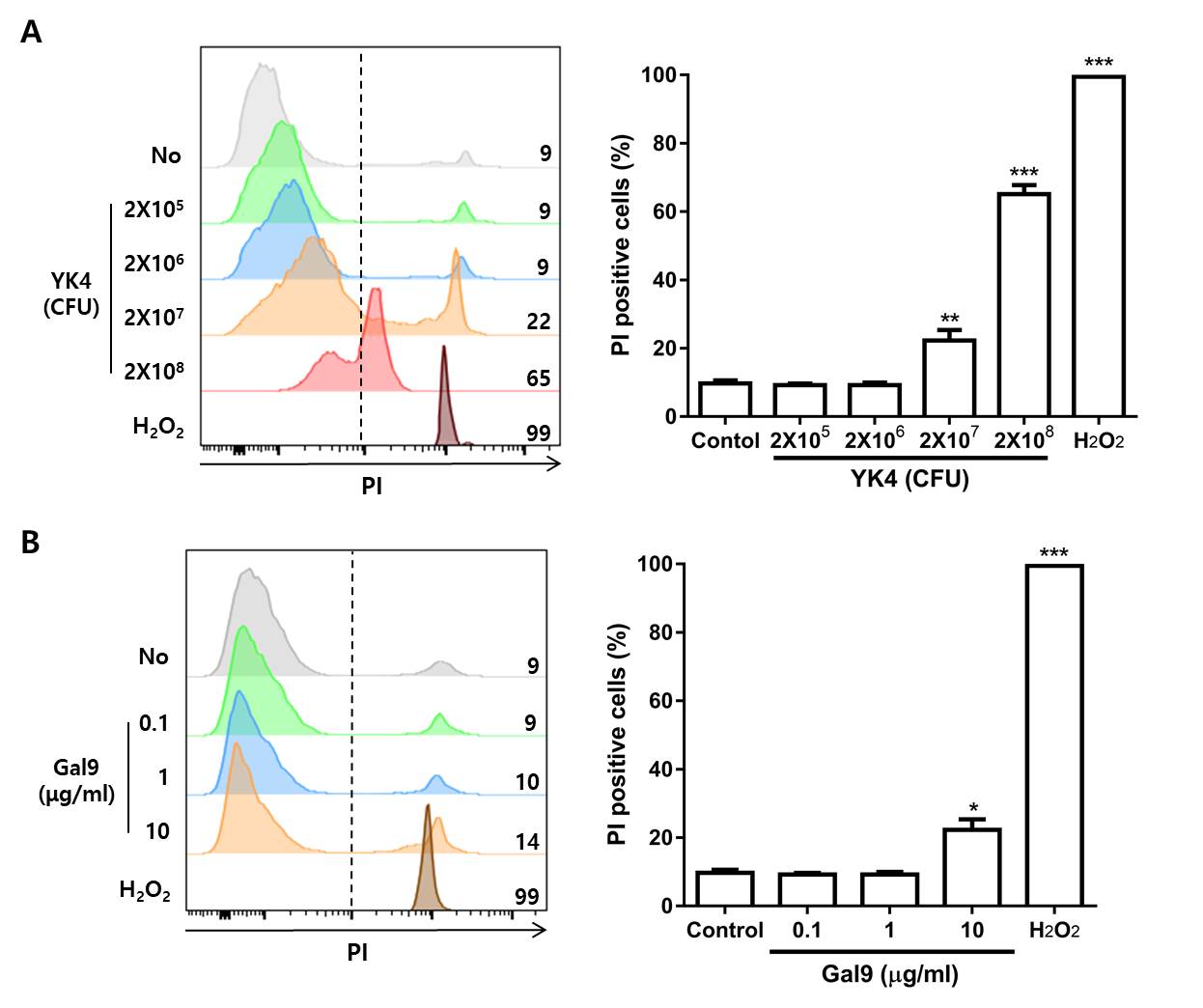

Supplement: Supplementary Figure 2 — Apoptosis of BMDCs treated with YK4 and galectin-9. BMDCs (2 × 10^5^ cells) were treated with various concentration of (A) YK4 and (B) galectin-9 for 24 h. Then, the percentage of apoptotic cells (the propidium iodide-positive fraction) was measured. H2O2 treatment was used as a positive control. A histogram of the results is shown. Data are representative of at least three experiments. *P < 0.05, **P < 0.01, ***P < 0.001 compared to the non-treated control group (Control) using Student's t-test. [file Image_2.JPEG]

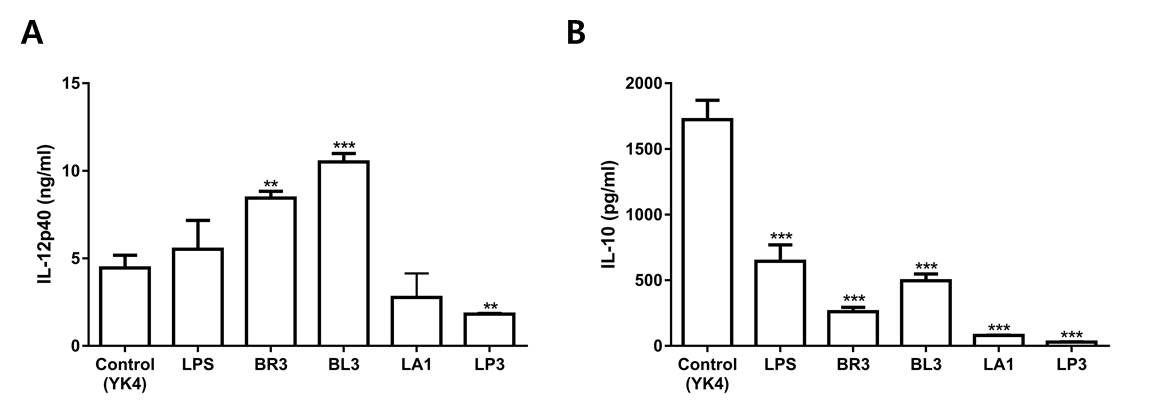

Supplement: Supplementary Figure 3 — Changes of cytokine secretion in BMDCs treated with each component of YK4. BMDCs were treated with 100 ng/ml of LPS or 2 × 10^6^ CFU of (YK4, BR3, BL3, LA1, LP3) for 24 h. The expression of (A) IL-12 and (B) IL-10 in the supernatants was measured by ELISA. Data are representative of at least three experiments. **P < 0.01, ***P < 0.001 compared to the YK4-treated group (Control) using Student's t-test. [file Image_3.JPEG]
